# Complete mitochondrial genome of *Agrostis stolonifera*: insights into structure, Codon usage, repeats, and RNA editing

**DOI:** 10.1186/s12864-023-09573-1

**Published:** 2023-08-18

**Authors:** Jiaxing Li, Yinglong Chen, Yaling Liu, Chen Wang, Ling Li, Yuehui Chao

**Affiliations:** 1 School of Grassland Science, Beijing Forestry University, Beijing, 100083 China; 2 UWA School of Agriculture and Environment, The UWA Institute of Agriculture, The University of Western Australia, Perth, WA 6001 Australia; 3 Inner Mongolia M-Grass Ecology And Environment (Group) Co., Ltd, Hohhot, 010010 China; 4 Mentougou District Bureau of Ecological and Environment of Beijing Municipality, Beijing, 102300 China

**Keywords:** *A. stolonifera*, Mitochondrial genome, Repeat-mediated recombination, RNA editing events

## Abstract

**Background:**

Plants possess mitochondrial genomes that are large and complex compared to animals. Despite their size, plant mitochondrial genomes do not contain significantly more genes than their animal counterparts. Studies into the sequence and structure of plant mitochondrial genomes heavily imply that the main mechanism driving replication of plant mtDNA, and offer valuable insights into plant evolution, energy production, and environmental adaptation.

**Results:**

This study presents the first comprehensive analysis of *Agrostis stolonifera*’s mitochondrial genome, characterized by a branched structure comprising three contiguous chromosomes, totaling 560,800 bp with a GC content of 44.07%. Annotations reveal 33 unique protein-coding genes (PCGs), 19 tRNA genes, and 3 rRNA genes. The predominant codons for alanine and glutamine are GCU and CAA, respectively, while cysteine and phenylalanine exhibit weaker codon usage biases. The mitogenome contains 73, 34, and 23 simple sequence repeats (SSRs) on chromosomes 1, 2, and 3, respectively. Chromosome 1 exhibits the most frequent A-repeat monomeric SSR, whereas chromosome 2 displays the most common U-repeat monomeric SSR. DNA transformation analysis identifies 48 homologous fragments between the mitogenome and chloroplast genome, representing 3.41% of the mitogenome’s total length. The PREP suite detects 460 C-U RNA editing events across 33 mitochondrial PCGs, with the highest count in the *ccmFn* gene and the lowest in the *rps7* gene. Phylogenetic analysis confirms *A. stolonifera*’s placement within the Pooideae subfamily, showing a close relationship to *Lolium perenne*, consistent with the APG IV classification system. Numerous homologous co-linear blocks are observed in *A. stolonifera*’s mitogenomes and those of related species, while certain regions lack homology.

**Conclusions:**

The unique features and complexities of the *A. stolonifera* mitochondrial genome, along with its similarities and differences to related species, provide valuable insights into plant evolution, energy production, and environmental adaptation. The findings from this study significantly contribute to the growing body of knowledge on plant mitochondrial genomes and their role in plant biology.

**Supplementary Information:**

The online version contains supplementary material available at 10.1186/s12864-023-09573-1.

## Background

Energy is a crucial requirement throughout the life cycle of eukaryotes, with mitochondria playing a key role in producing biological energy (ATP) [1]. Initially believed to be independent organisms in a symbiotic relationship with larger cells, mitochondria have since lost their ability to survive independently due to the transfer of their original genes into the host genome [2, 3]. Nevertheless, the remaining mitochondrial DNA is essential for processes like respiration, DNA replication, transcription, tRNA synthesis, and other organelle functions [4–6].

Despite their similar functions, plant and animal mitochondrial genomes differ significantly in size [7, 8]. Animal mitochondrial genomes are typically around 16.5 kb with few introns and non-coding regions, whereas plant mitochondrial genomes are larger, ranging from 200 to 2,000 kb, containing abundant repeat sequences, AT-rich non-coding regions, and large introns and non-coding sequences [9]. Furthermore, plant mitochondrial genomes contain significant amounts of short nuclear and chloroplast genomic sequences and uniquely undergo RNA editing, a process not found in mammals [10]. Despite these differences, plants do not encode more genes in their mitochondrial genomes than animals do.

The first mitochondrial genome sequence of a terrestrial plant was reported in 1992, and since then, numerous higher plant mitochondrial genomes have been sequenced and analyzed for structure [11, 12]. As of March 2023, 471 land plant mitochondrial genomes have been deposited in the National Center for Biotechnology Information.

*Agrostis stolonifera*, belonging to the genus Shearling of the family Gramineae, is a crucial cool-season turfgrass. It is widely distributed in temperate Eurasia and North America due to its prostrate growth and is the most tolerant of all cool-season turfgrasses to continuous low mowing in cold, wet, and transitional climates [13, 14]. Additionally, *A. stolonifera* is an allotetraploid plant (2n = 4x = 28) [15, 16]. Polyploid plants can combine multiple chromosome sets in a single nucleus and double the number of alleles at each locus, resulting in various genetic changes, such as chromosomal recombination, sequence elimination, gene silencing, activation, and expression levels, which can lead to different evolutionary directions [17, 18]. Heterozygous tetraploid plants, in comparison to homozygous tetraploid plants, come from different parents and rely on chromosomal recombination for the generation of new variants and phenotypes in their offspring [19]. These heterozygous tetraploid plants can experience frequent chromosome exchange, exhibit various types of chromosome rearrangements, and demonstrate greater adaptive capacity than homozygous tetraploids [20]. At the epigenetic level, hetero-tetraploid plants may induce effects such as DNA methylation, transposon activation, and changes in RNA editing sites [21]. With the development of transcriptome sequencing technology, more scholars have been able to more accurately identify the internal structure and functional changes of genes in polyploid plants, thus deepening our understanding of polyploid gene expression changes. Recently, the complete chloroplast and nuclear genome sequences have been discovered by a large number of studies in *A. stolonifera* [22, 23]. However, the complete mitochondrial genome sequence of *A. stolonifera* is still unknown.

This study represents the first investigation of the complete sequence and structure of the mitochondria genome from *A. stolonifera*. The study includes functional annotation, codon usage analysis, repeat sequence identification, comparative mitochondrial genome analysis, gene transfer, and RNA edition analysis. These data expand the genetic information available and provide new insights into the genetic improvement of *A. stolonifera*.

## Results

### Structure features and annotation of *A. stolonifera* Mitogenome

**Fig. 1:**
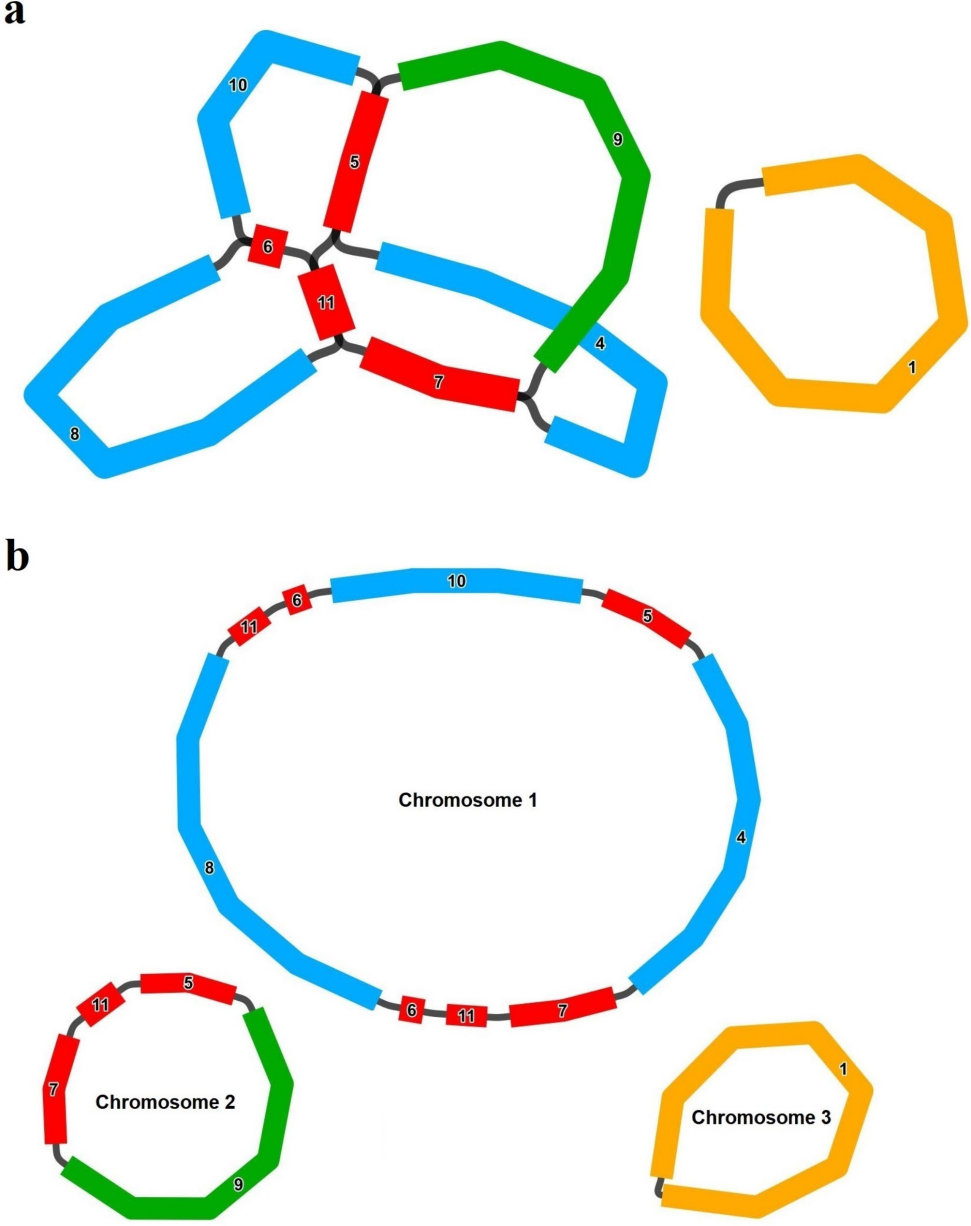
Structure features of *A. stolonifera* mitogenome. **(a)** Branched conformation of *A. stolonifera* mitogenome. **(b)** Three circular molecules of *A. stolonifera* mitogenome

The main architecture of the *A. stolonifera* mitochondrial genome is branched. After exclusion of duplicated regions from the Nanopore sequencing data, three contiguous sequences (Chromosome 1–3) were obtained totaling 560,800 bp with a GC content of 44.07%. Chromosome 1 was 300,195 bp in length, Chromosome molecule 2 was 139,595 bp, and Chromosome 3 was 121,010 bp, with GC contents of 44.03%, 44.33%, and 43.88%, respectively (Fig. 1).

**Fig. 2:**
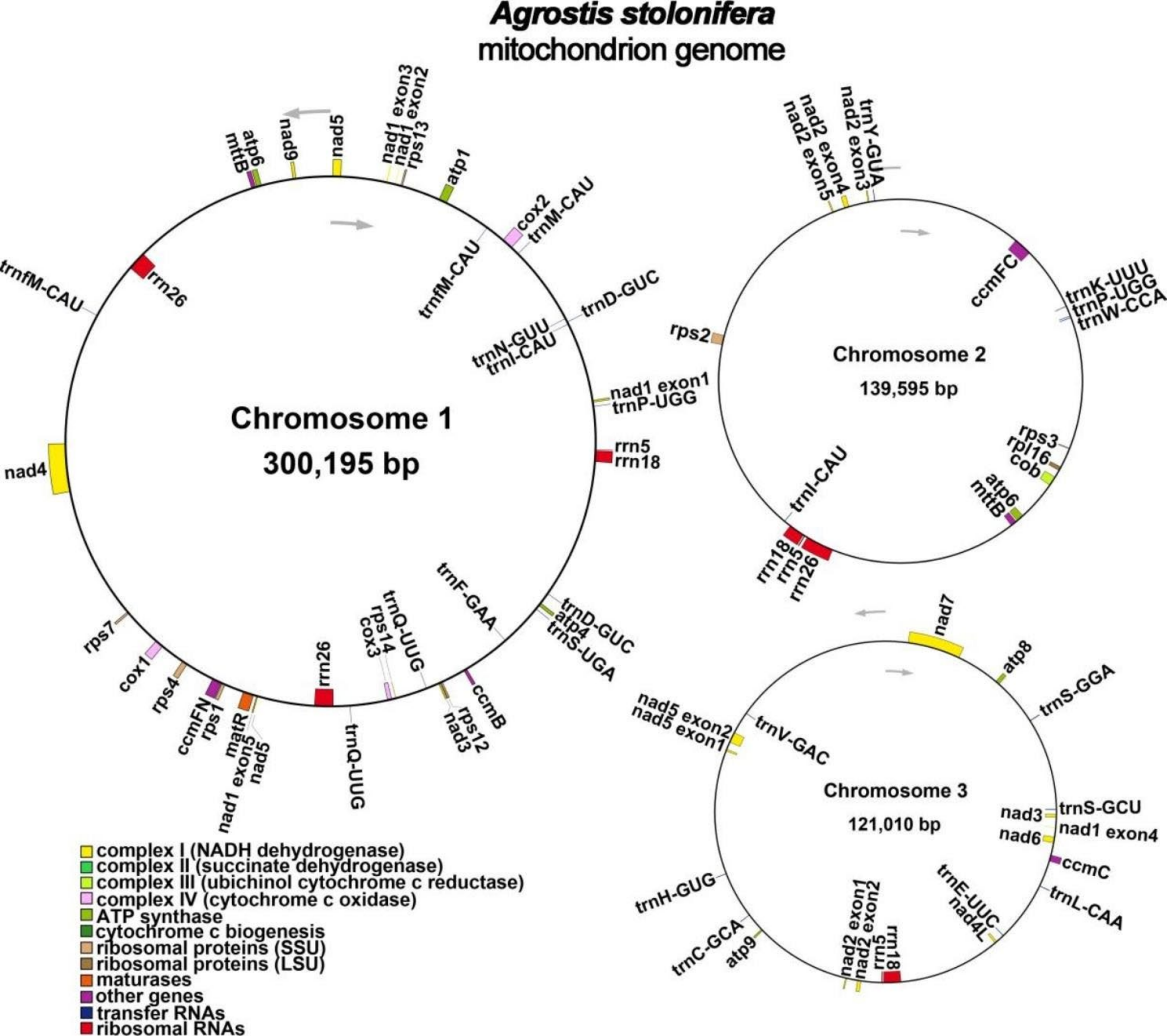
* A. stolonifera* mitogenome gene map. Genes located inside the circle are transcribed in a clockwise direction, while those outside the circle are transcribed counterclockwise. The inner circle features a dark gray region which depicts the GC content

The *A. stolonifera* mitochondrial genome was annotated with 33 unique PCGs, consisting of 24 mitochondrial core and 9 non-core genes, as well as 19 tRNA and 3 rRNA genes (Table 1; Fig. 2). Of the 24 unique mitochondrial core genes, 5 are related to ATP synthesis (*atp1*, *atp4*, *atp6*, *atp8* and *atp9*), 9 are NADH dehydrogenase genes (*nad1*, *nad2*, *nad3*, *nad4*, *nad4L*, *nad5*, *nad6*, *nad7* and *nad9*), 4 are cytochrome C reductase genes (*ccmB*, *ccmC*, *ccmFc*, and *ccmFn*), 3 are cytochrome c oxidase genes (*cox1*, *cox2*, and *cox3*), 1 is a transport membrane protein gene (*mttB*), 1 is a maturases gene (*matR*), and 1 is a ubiquinol-cytochrome C biogenesis gene (*cob*). Non-core genes include 1 large subunit ribosomal gene (*rpl16*) and 8 small subunit ribosomal genes (*rps1*, *rps2*, *rps3*, *rps4*, *rps7*, *rps12*, *rps13*, and *rps14*).

**Table 1:** Gene annotation of the *A. stolonifera* mitochondrial genome

Group of genes	Name of genes
ATP synthase	*atp1*, *atp4*, *atp6* (×2), *atp8*, *atp9*
NADH dehydrogenase	*nad1*, *nad2*, *nad3* (×2), *nad4*, *nad4L*, *nad5*, *nad6*, *nad7*, *nad9*
Cytochrome c biogenesis	*cob*
Ubiquinol cytochrome c reductase	*ccmB*, *ccmC*, *ccmFC*, *ccmFN*
Cytochrome c oxidase	*cox1*, *cox2*, *cox3*
Maturases	*matR*
Transport membrane protein	*mttB* (×2)
Large subunit of ribosome	*rpl16*
Small subunit of ribosome	*rps1*, *rps2*, *rps3*, *rps4*, *rps7*, *rps12*, *rps13*, *rps14*
Ribosome RNA	*rrn5* (×3), *rrn18* (×3), *rrn26* (×3)
Transfer RNA	*trnC-GCA*, *trnD-GUC* (×2), *trnE-UUC*, *trnF-GAA*, *trnfM-CAU* (×2), *trnH-GUG*, *trnI-CAU* (×2), *trnK-UUU*, *trnL-CAA*, *trnM-CAU*, *trnN-GUU*, *trnP-UGG* (×2), *trnQ-UUG* (×2), *trnS-GCU*, *trnS-GGA*, *trnS-UGA*, *trnV-GAC*, *trnW-CCA*, *trnY-GUA*

**Note**: The numbers in brackets represent the copy number of genes. For example, (×2) means there are two copies

### Codon usage analysis of PCGs in *A. stolonifera*

**Fig. 3:**
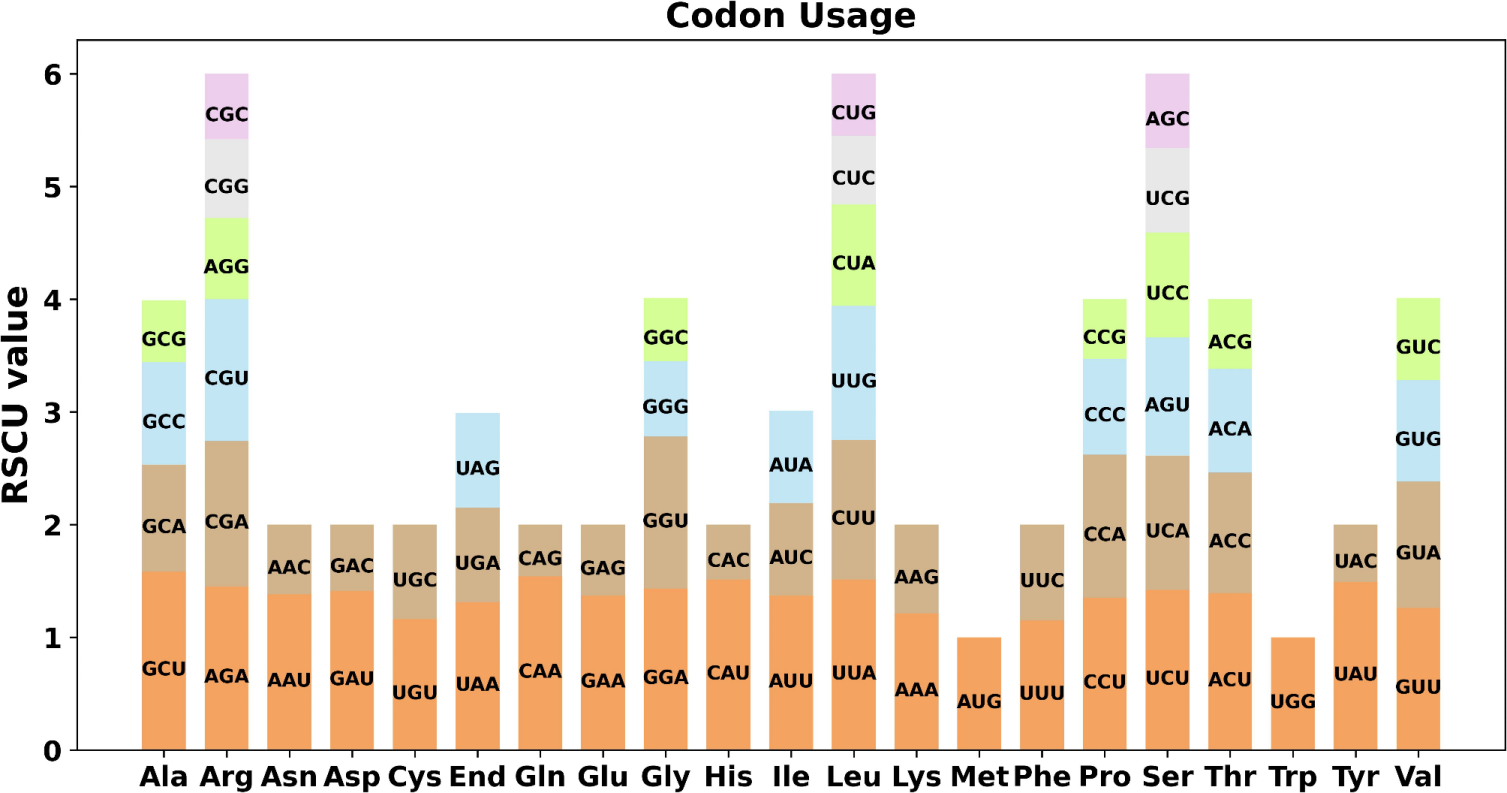
Analysis of codon usage bias in *A. stolonifera* mitochondrial genomes. X-axis, codon families; Y-axis, the relative synonymous codon usage (RSCU) value. RSCU measures the likelihood of a specific codon being used among synonymous codons that encode the same amino acid and values greater than 1 indicate a higher frequency of usage for the codon

The codon usage of 33 unique PCGs from *A. stolonifera* was analyzed, and those with a relative synonymous codon usage (RSCU) greater than 1 were considered to be preferentially used by amino acids. The RSCU values for the start codons AUG (Met) and UGG (Trp) were both equal to 1, while a general codon usage preference was observed for the mitochondrial PCGs (Fig. 3; Supplementary Table 1). GCU had the highest RSCU value (1.58), followed by CAA (1.54), indicating high frequency usage for alanine (Ala) and glutamine (Gln), respectively. It is worth noting that the maximum RSCU values for cysteine (Cys) and phenylalanine (Phe) were less than 1.2, indicating a lack of strong codon usage bias. The frequent usage of A or U nucleotide in the third codon position, compared to other nucleotides, was also observed. This is a common characteristic in the mitogenomes of land plant species.

### Repeat sequence analysis of mitochondrial genomes

**Fig. 4:**
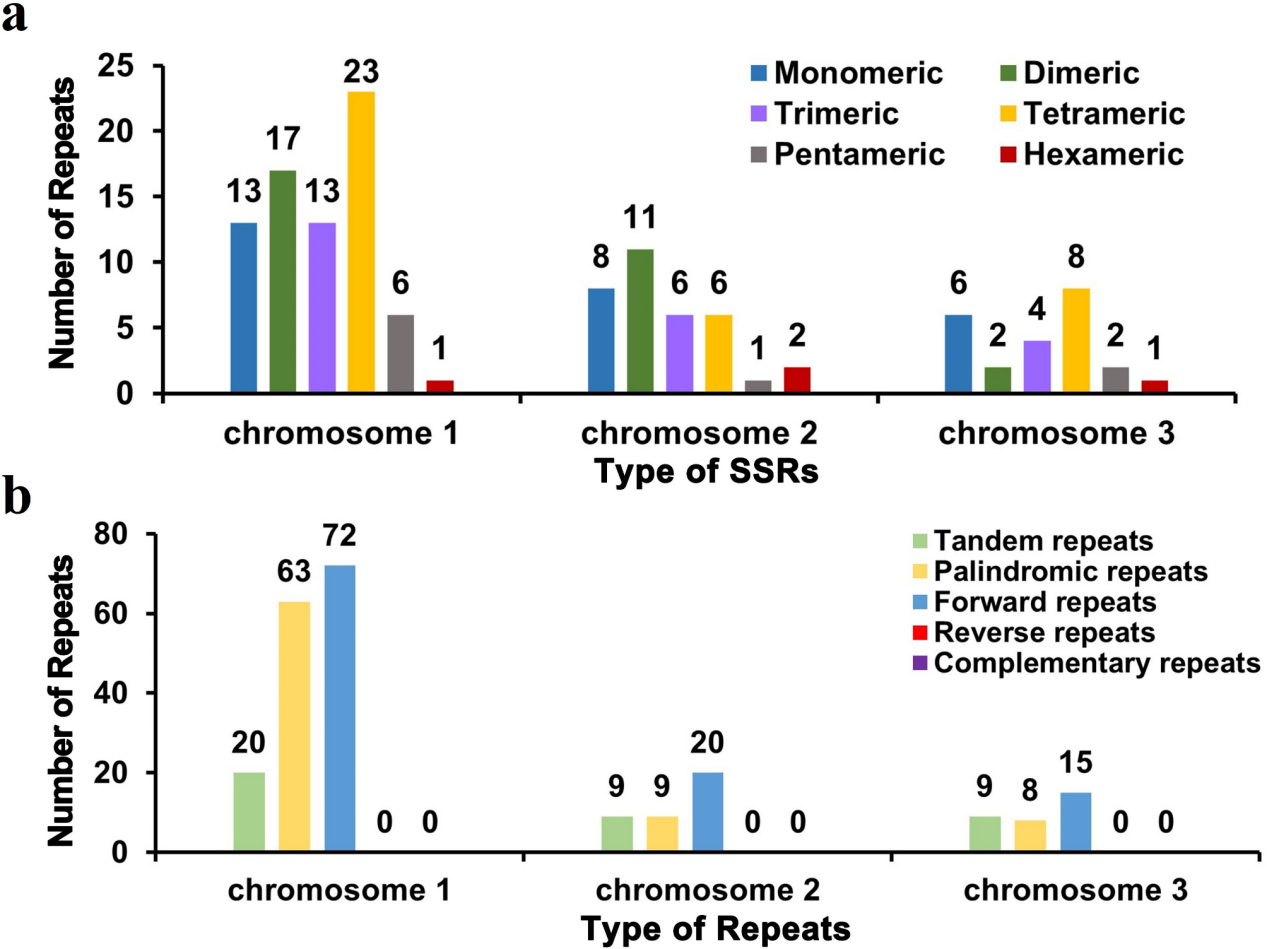
Repeat sequence analysis of the *A. stolonifera* mitochondrial genome. (**a**) The x-axis represents the type of SSRs while the y-axis represents the number of repeats. Each colored legend represents a different type of SSR: purple for monomer, yellow for dimer, blue for trimer, green for tetramer, gray for pentamer, and red for hexamer SSRs. (**b**) The x-axis displays the type of repeats, and the y-axis displays the number of repeats. The green, red, and blue legends correspond to tandem, palindromic, and forward repeats, respectively. Notably, neither reverse nor complementary repeats were identified in the mitochondrial genome under investigation

The mitogenome of *A. stolonifera*’s Chromosome 1 was found to contain 73 simple sequence repeats (SSRs), 41.10% of which were monomeric and dimeric. The most frequent monomeric SSR was an A repeat monomer, occurring 7 times and making up 53.85% of the total. The most common dimeric SSR was UA/UC, accounting for 47.06%. Chromosome 1 also contained one hexameric SSR. The repeat analysis showed 20 tandem repeats in Chromosome 1, ranging from 14 to 65 bp, and 135 non-tandem repeats of ≥ 30 bp length. Of these, 63 were palindromic and 72 were forward repeats, with no reverse or complementary repeats. The largest palindromic and forward repeats measured 289 bp and 10,577 bp, respectively. Chromosome 2 contained 34 SSRs, of which 55.88% were monomeric and dimeric. The most prevalent monomeric SSR was a U repeat monomer, which occurred 6 times and made up 75% of the 8 monomeric SSRs. Two hexameric SSRs were found in Chromosome 2. The repeat analysis showed 9 tandem repeats in Chromosome 2, ranging from 18 to 65 bp, and 29 non-tandem repeats of ≥ 30 bp length. Of these, 9 were palindromic and 20 were forward repeats, with no reverse or complementary repeats. The largest palindromic and forward repeats measured 62 and 436 bp, respectively. Chromosome 3 contained 23 SSRs, with 34.78% being monomeric and dimeric. The most prevalent monomeric SSR was an A repeat monomer, which occurred 4 times and made up 66.67% of the total. Chromosome 3 also contained one hexameric SSR. The repeat analysis showed 9 tandem repeats in Chromosome 3, ranging from 17 to 65 bp, and 23 non-tandem repeats of ≥ 30 bp length. Of these, 8 were palindromic and 15 were forward repeats, with no reverse or complementary repeats. The largest palindromic and forward repeats measured 46 and 76 bp, respectively (Fig. 4).

### DNA transfer from Chloroplast to Mitochondrion

**Fig. 5:**
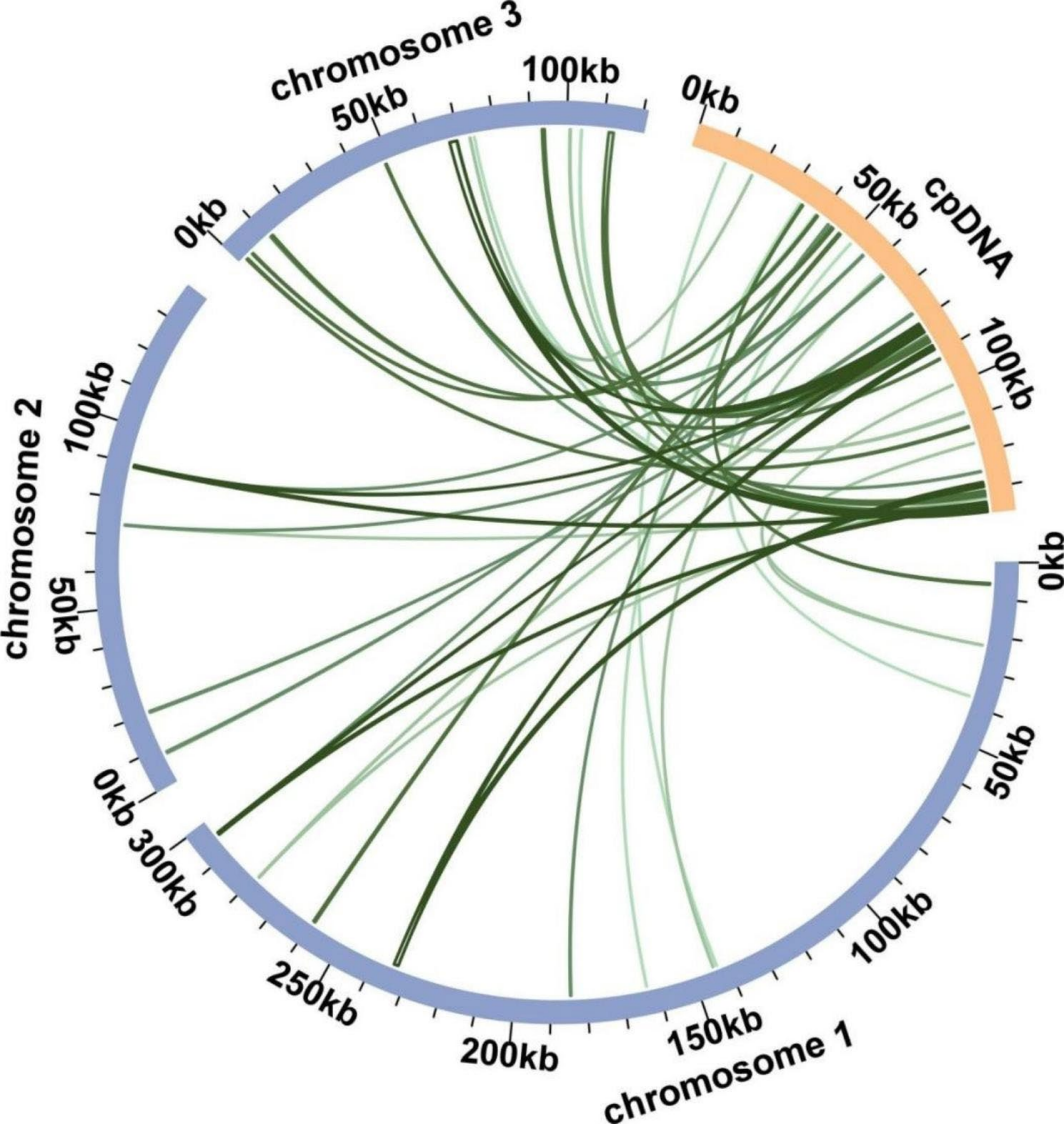
The gene transfers that occurred between the chloroplast and mitochondrial genomes of *A. stolonifera*. The blue and orange arcs denote the mitochondrial and chloroplast genomes, respectively, while the green lines connecting the arcs represent homologous genome segments that were transferred between the two organelles

The chloroplast genome was sequenced, assembled and annotated (Supplementary Fig. S1), which was used for DNA transformation analysis. A total of 48 fragments were found in the mitogenome that were homologous to the chloroplast genome, accounting for 3.41% of the mitogenome’s total length (19,114 bp; Fig. 5; Supplementary Table S2). The two longest fragments were 1 and 2, each measuring 4760 bp. Upon annotation, these homologous sequences revealed 12 complete genes, including 1 PCG (*rpl2*) and 11 tRNA genes (*trnC-GCA*, *trnF-GAA*, *trnH-GUG*, *trnI-CAU*, *trnL-CAA*, *trnM-CAU*, *trnN-GUU*, *trnP-UGG*, *trnS-GGA*, *trnV-GAC*, and *trnW-CCA*).

### RNA editing events in Mitochondrion

**Fig. 6:**
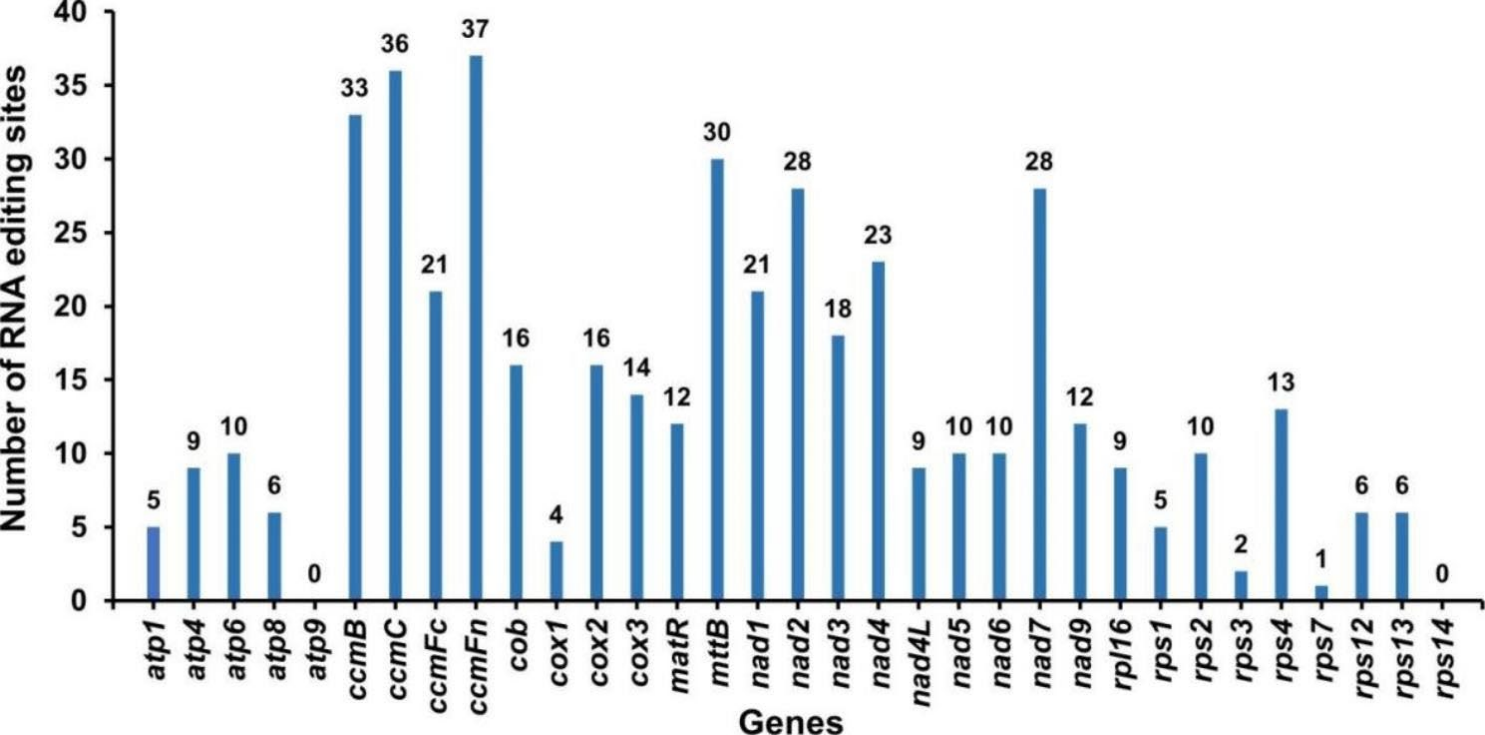
The Number of RNA editing sites predicted in PCGs of *A. stolonifera* mitochondrial genome

The PREP suite was utilized to predict RNA editing events with a cutoff value of 0.2. As a result, 460 RNA editing events were identified in 33 mitochondrial PCGs (Fig. 6). The *ccmFn* gene contained the highest number of RNA editing sites at 36, while the *rps7* gene had the lowest number of events with only 1. No RNA editing events were found in the *atp9* and *rps14* genes, and all of the identified events were of the C-U type (Supplementary Table S3).

**Fig. 7:**
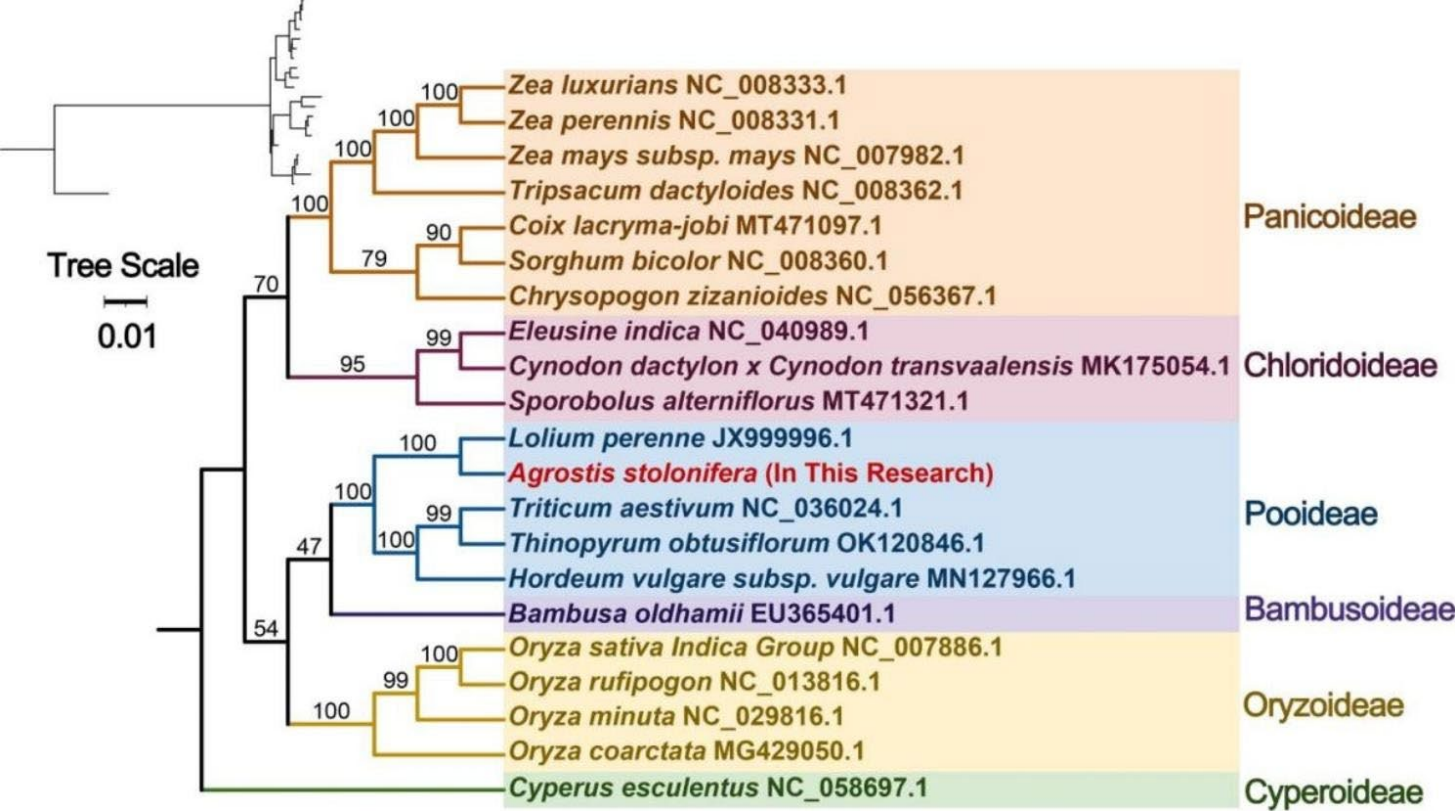
The phylogenetic relationships of *A. stolonifera* with other closely related species.The Neighbor-Joining tree was constructed based on the sequences of 22 conserved PCGs. Colors indicate the families that the specific species belongs

Mitochondrial DNA sequences from a total of 21 species from 6 subfamilies of Poaceae were obtained from the NCBI database. A phylogenetic analysis was performed based on 22 conserved mitochondrial PCGs (*atp4*, *atp6*, *ccmB*, *ccmC*, *ccmFc*, *cob*, *cox1*, *cox2*, *cox3*, *nad1*, *nad2*, *nad3*, *nad4*, *nad5*, *nad6*, *nad7*, *rpl16*, *rps3*, *rps4*, *rps7*, *rps12* and *rps13*). The results revealed that *A. stolonifera* is part of the Pooideae subfamily in the Poaceae family and is closely related to *Lolium perenne* (Fig. 7). This supports the consistency of the phylogenetic tree obtained from the mitochondrial PCGs with the APG IV classification system.

**Fig. 8:**
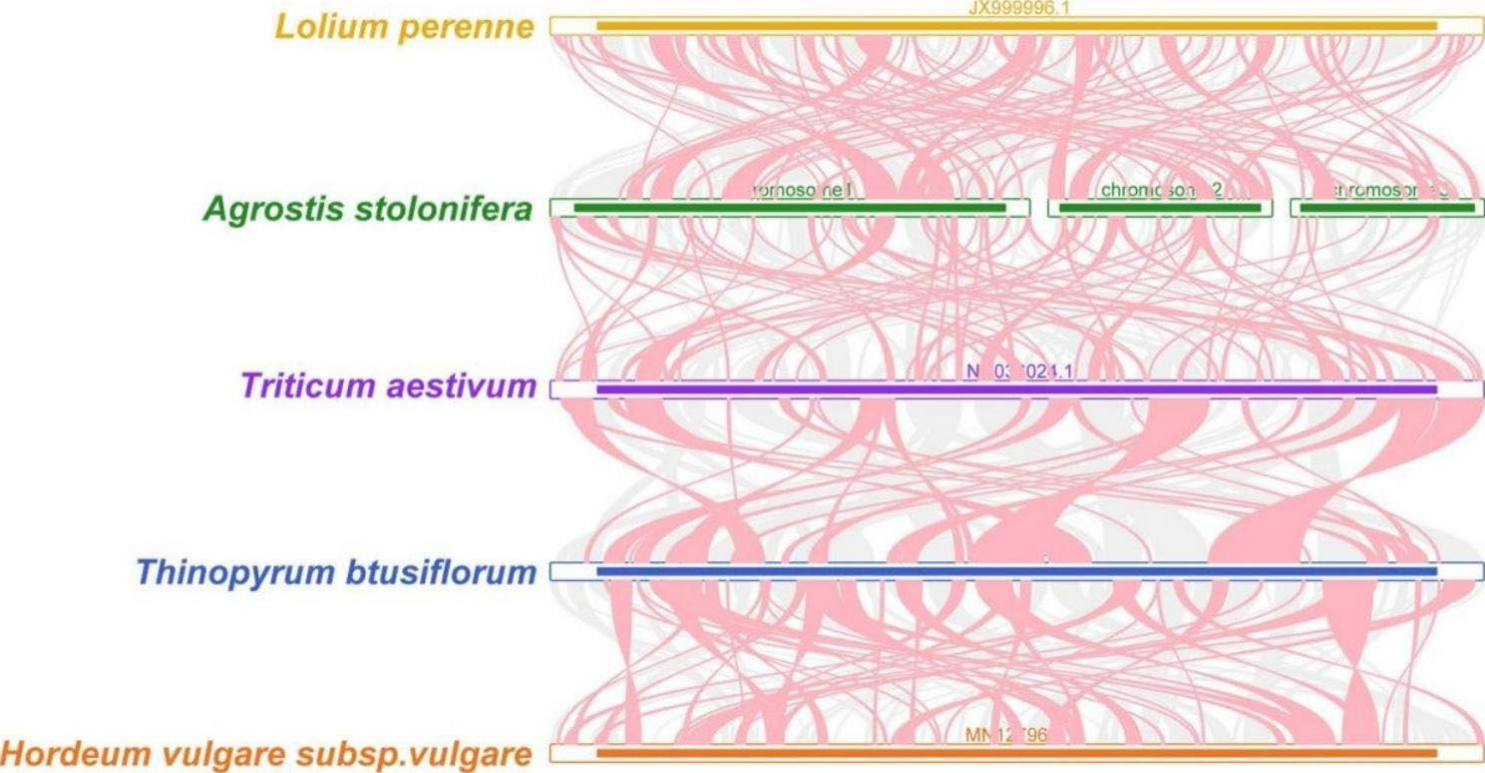
Mitochondrial genome Multiple Synteny Plot of *A. stolonifera* and closely related species.The bars on the graph indicate the mitochondrial genomes of the species, while the ribbons depict the homologous sequences between adjacent species. The red areas highlight where inversions occurred, while the gray areas indicate regions with strong homology

The analysis of the mitogenomes of *A. stolonifera* and four closely related species, including *Lolium perenne*, *Triticum aestivum*, *Hordeum vulgare*, and *Thinopyrum obtusiflorum*, revealed the presence of a large number of homologous co-linear blocks (Fig. 8). However, the length of these blocks was relatively small, with the largest block measuring 12,998 bp in length and exhibiting 98.731% identity between chromosome 1 of the *A. stolonifera* and *Lolium perenne* mitogenomes. The arrangement of the co-linear blocks differed among individual mitogenomes, suggesting that the *A. stolonifera* mitogenome has undergone extensive genomic rearrangements in comparison to its closely related species and has an extremely unconserved structure. Additionally, certain regions of the *A. stolonifera* mitogenome exhibited no homology to the other species, demonstrating their exclusive presence in this mitogenome.

## Discussion

Mitochondria serve as the powerhouse of energy, playing a pivotal role in producing the energy required to maintain cellular life [24]. Compared to animal mitochondrial genomes, plant mitochondrial genomes are characterized by their complexity. Animal mitochondrial genomes typically range from 10 to 20 kb, whereas those of plants range from 190 kb to 11.3 Mb (commonly 400-800 kb) [25]. Plant mitochondria genomes contain only a few coding genes with highly conserved sequences that are sparsely distributed [26]. Although circular maps are commonly used to represent them during mitosis, recent studies have shown that they are unlikely to exist as a single deoxyribonucleic molecule [27–29]. Advances in sequencing technologies, including Illumina’s second-generation sequencing and Nanopore’s third-generation sequencing, have enabled researchers to explore the complex structure of these genomes more accurately [30–33]. This has led to a better understanding of their biological replication and recombination mechanisms and their unique functional and evolutionary processes. In this study, we sequenced *A. stolonifera*, a member of the Gramineae family, which has a multibranched primary structure containing three looped contigs, similar to the *Populus deltoides* mitogenome [34]. This is unlike the mitogenomes of a sugarcane cultivar [35], *Oryza sativa* [36], *Elymus sibiricus* [37], and most other graminid mitochondrial genomes, which have single loops. The comparative analysis of DNA sequences of closely related species has revealed DNA repair can occur between closely related species through homologous recombination and exchange of nucleotide sequences at non-homologous ends. This process can result in the loss of coding frames or regulation of gene expression, and if not repaired promptly and accurately, it can lead to chromosome loss and recombination in plants, ultimately resulting in structural and functional differences in genes. However, the mechanism underlying the coexistence of these three circular molecules in *A. stolonifera* is still unclear and requires further investigation. Additionally, the *A. stolonifera* genome is GC-rich, which could serve as an indicator to determine the species [38, 39]. With a total length of 560,800 bp and 44.07% GC content, *A. stolonifera* is highly similar to its fellow graminae Lolium perenne (GenBank, JX 999,996) [40] and Hordeum vulgare (GenBank, AP017300 and AP017301) [41], which have GC contents of 44.1% and 44.2%, respectively. The GC content of the entire mitochondrial genome is remarkably similar and consistent with the conservation observed in higher plants.

The eukaryotic genome contains 64 codons that code for 20 different amino acids and three stop codons. With the exception of Met and Trp, all amino acids are encoded by two to six synonymous codons. The preference for the utilization of these synonymous codons is determined by several factors, including the abundance of tRNA, the mutational bias of the gene chain, gene expression level, gene length, and GC composition. The codon preference analysis of 33 unique PCGs of *A. stolonifera* mitochondria and the use of codons by individual amino acids are shown in Fig. 3 .The PCGs of *A. stolonifera* mitochondria typically begin with ATG start codons and preferentially end with A or U in their stop codon. This result is similar to the codon preference of higher angiosperms [42–44] .

In contrast to animal and yeast mitochondria, plant mitochondria contain abundant introns and repetitive elements in their mitochondrial repetitive sequences, accounting for up to 90% of the entire mt-genomes. Repeats play a significant role in mitochondrial intergenic sequences [45, 46]. Plant mitochondrial large repeating segments, over 1,000 bp, frequently undergo reciprocal recombination, which not only subdivides the genome, increasing recombination viability, but also generates the coexistence of small and large loop structures. Frequent recombination of repetitive sequences contributes to this phenomenon in mitochondria [47, 48]. We hypothesize that the multiple loop structure of *A. stolonifera*, as shown in Fig. 2, is correlated with its frequent repeat sequences. Recombination variation in *A. stolonifera* is significantly different and mainly concentrated in the mitotic genome of chromosome 1, which contains 73 variations of SSRs. A recent study suggests that these homologous recombination patterns contribute to reproductive diversity in higher plants, particularly through homologous recombination repair [49]. Although these rearrangements can lead to developmental issues or lethality, they may also result in a beneficial phenotype [50, 51]. Studies on the maintenance of the Arabidopsis mitochondrial *SSB1* and *SSB2* genes, which are involved in the ABA signaling pathway during seed germination and play roles in mitochondrial replication and homologous recombination, revealed that they negatively regulate mtDNA replication [52, 53]. Additionally, during mitochondrial evolution, some chloroplast fragments migrated into the mitochondrial genome, with the length and sequence similarity of the migrated fragments varying over time. The growth and development of plant leaves and roots at different stages can cause fragments of various lengths from the chloroplast genome to migrate into the mitochondrial genome. As a result, higher plant mitochondrial genomes contain sequences that are homologous to chloroplast DNA, facilitating the movement of genetic material within the organism. In our study, we identified 48 mitochondrial genome fragments with a total length of 19,114 bp that were homologous to the chloroplast genome, including 12 complete genes. This finding demonstrates the existence of gene transfer between chloroplasts and mitochondria [54].

RNA editing, a deamination reaction, is essential for gene expression in higher plant mitochondrial genomes and occurs through a post-transcriptional process. Studying RNA modification target sites further advances our understanding of the molecular mechanisms underlying plant gene expression in both mitochondrial genomes and chloroplast genomes [55]. Previous studies have reported this phenomenon, such as 421 RNA editing sites in 26 genes of *A. truncatum* [56], 457 RNA editing sites in 36 genes of *Rhopalocnemis phalloides* [57], and 597 RNA editing sites in 35 genes of *Ipomoea batatas* Lam [58]. In our study, we identified 460 RNA editing sites in 33 PCGs of *A. stolonifera* mitochondria using online point prediction, with all of them being C-U RNA editing. This C-U RNA editing event predominantly occurs at the second codon position and is mostly fully edited, enabling the regulation of RNA editing sites in *A. stolonifera*. This process enhances the homology of mitochondrial protein sequences among different species, promotes plant growth and development, and could be exploited for use in other species.

To clarify the phylogeny of representative taxa of *A. stolonifera* based on mitochondrial genomic information and establish well-defined taxonomic relationships among them, we constructed a phylogenetic tree using PCGs. The analysis revealed that *A. stolonifera* is closely related to Lolium perenne, which is supported by their similar GC content. This finding further corroborates the congruence of the phylogenetic tree derived from mitochondrial PCGs with the APG IV classification system. By examining the arrangement of different co-linear blocks in individual mitochondrial genomes, we discovered that the *A. stolonifera* mitochondrial genome has experienced extensive genomic rearrangements, resulting in a highly variable structure compared to its close relatives. This allows for the evolution and diversification of the mitochondrial genome. Some regions of the *A. stolonifera* mitochondrial genome do not exhibit homology with other species, indicating their unique presence in this mitotic genome. This significant discovery has implications for future studies on the genetics, growth, and development of *A. stolonifera*.

## Conclusions

The findings of this study provide new insights into the evolution, energy production, and adaptation of plants to the environment. The discovery of RNA editing in the mitochondrial PCGs of *A. stolonifera* expands our understanding of the unique genetic features of plant mitochondria. The identification of SSRs and homologous co-linear blocks in the mitogenomes of *A. stolonifera* and related species paves the way for future genetic improvement studies. Overall, this study highlights the importance of understanding the mitochondrial genome in both basic and applied plant sciences.

## Materials and methods

### Plant materials, DNA extraction and *De novo* sequencing

The seeds of *A. stolonifera* (cv. ‘Penn A-4’) were bought from Barenbrug USA (Oregon, USA) and stored at School of Grassland Science, Beijing Forestry University, which were cultivated in a light culture chamber (26/20℃, 16 h light/8 h dark cycle with 50% humidity). Leaves from a 30-day-old seedling were harvested and used for DNA extraction by CTAB method. The DNA quantity and quality were checked using the Nanodrop and Qubit for library construction and sequencing. The mitochondrial genome sequencing was performed on BGI and Nanopore platforms.

### Sequence assembly and annotation

The GetOrganelle software (v1.7.5) with default parameters was utilized to assemble *A. stolonifera* mitochondrial genome from short-read data, and the Bandage software was used to visualize the mitochondrial genome and remove single extended fragments from chloroplast and nuclear genomes. To make mitochondrial genome sequence more accurate, Flye software was employed to assembly and a graphical representation of the mitochondrial genome was generated with long-read data. The bwa software was used to compare short-read data to the graphical representation of the mitochondrial genome obtained from long-read data. Repeated sequences were excluded from the second-generation assembly results to ensure consistency between the short-read and long-read assembly results, resulting in the final mitochondrial genome of *A. stolonifera*.

*Lolium perenne* (JX999996.1) and *Liriodendron tulipifera* (NC_021152.1) were selected as the reference genomes for annotation. The Geseq software [59] was employed to annotate the coding genes of the mitochondrial genome from *A. stolonifera*. The tRNA genes were annotated using tRNAscan-SE software [60], while the rRNA genes were annotated using BLASTN software [61]. To ensure accuracy, the annotation errors in the mitochondrial genome were manually corrected using Apollo software [62].

### Analysis of codon usage bias and repeated elements

The codon usage bias in various species and organisms differs significantly. It is believed that this bias is a result of prolonged evolutionary selection. The protein-coding sequences were extracted using Phylosuite software [63] and the relative synonymous codon usage (RSCU) values of the amino acid composition of protein coding genes from mitochondrial genome were determined using MEGA (v7.0) software. Simple sequence repeats (SSRs) were identified using the MISA software (https://webblast.ipk-gatersleben.de/misa/) [64]. Tandem repeats and non-tandem repeats in the mitochondrial genome were analyzed using TRF (https://tandem.bu.edu/trf/trf.unix.help.html) [65] and REPuter (https://bibiserv.cebitec.uni-bielefeld.de/reputer/) [66].

### DNA transfer and RNA editing events

The chloroplast genome was assembled using GetOrganelle [67] and annotated using CPGAVAS2 [68]. Homologous fragments were analyzed with BLASTN [61], and the results were visualized using the package RCircos [69]. The prediction of RNA editing events was based on the PREP suit website (http://prep.unl.edu/cgi-bin/mt-input.pl) [70].

### Phylogenetic and synteny analysis

The mitochondrial genome sequences of closely related plant species were obtained from the NCBI website, and common genes were extracted using PhyloSuite software [63]. Multiple sequence alignment analysis was performed using MAFFT [71] with a bootstrap value of 1000, and phylogenetic analysis was conducted using MRBAYES [72]. The final results of the phylogenetic analysis were then visualized using ITOL software [73].

To investigate the collinearity relationship of the mitogenome between various popular species, we used BLASTN [61] to align the nucleotides of the *A. stolonifera.*mitogenome with those of closely related plant species. Homologous sequences with a length of over 500 bp were then utilized to generate a multiple synteny plot of *the A. stolonifera* mitogenome in comparison to closely related species using MCscanX [74].

### Electronic supplementary material

Below is the link to the electronic supplementary material.

Supplementary Material 1

Supplementary Material 2

Supplementary Material 3

Supplementary Material 4

## Data Availability

The genome sequence data for the chloroplast and mitochondria of *A. stolonifera* have been uploaded to the NCBI database under Accession Numbers OQ695464 and OQ695465, respectively.
