# Supplementary figures and images for: Complete mitochondrial genome of Agrostis stolonifera: insights into structure, Codon usage, repeats, and RNA editing
Source: BMC Genomics. 2023 Aug 18;24:466. doi: 10.1186/s12864-023-09573-1 (PMC10439588; doi:10.1186/s12864-023-09573-1)

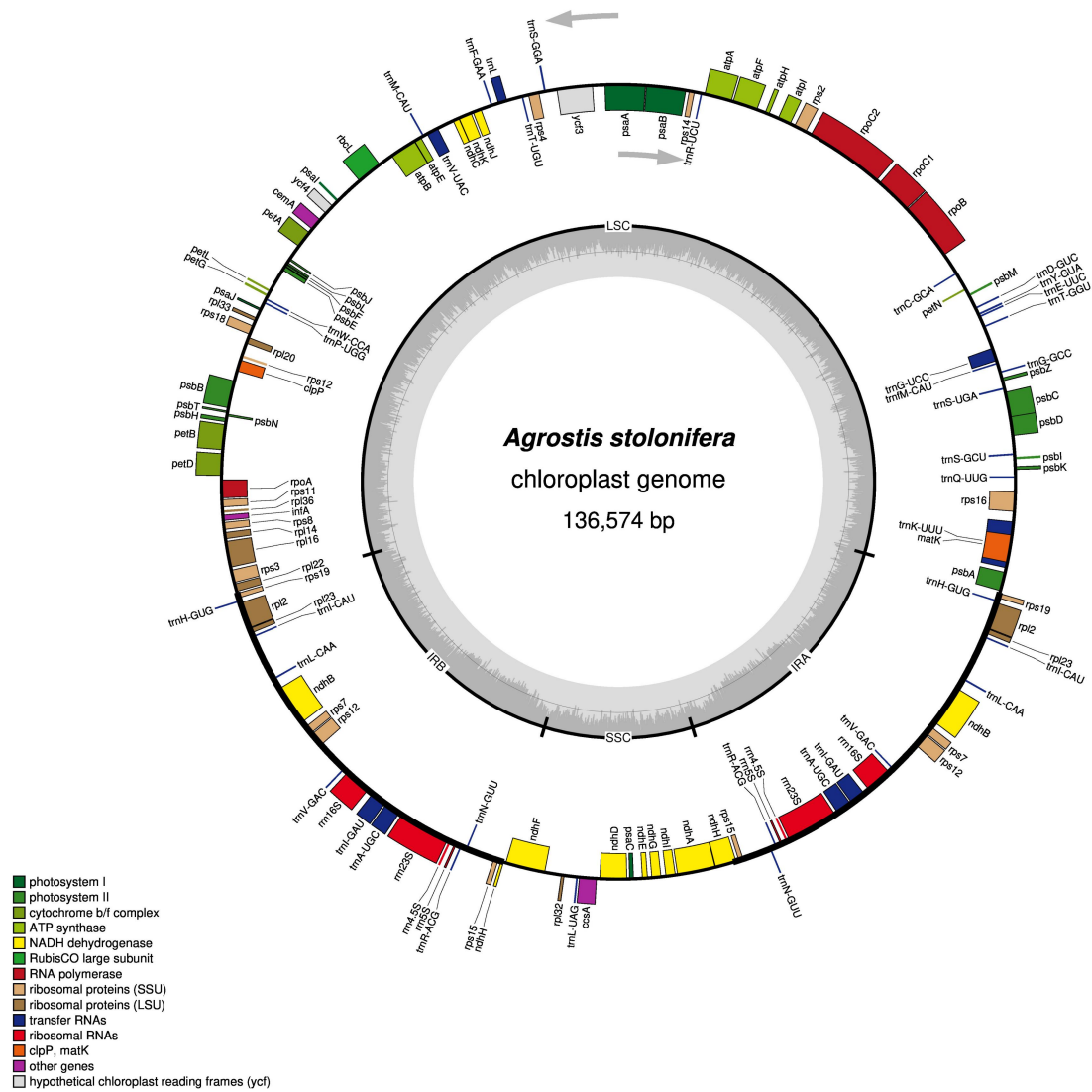

Figure. S1 Chloroplast genome map of *A. stolonifera*.

Supplement: Supplementary file 4 — Supplementary Material 4 [file 12864_2023_9573_MOESM4_ESM.pdf]
